# Genetic Susceptibility to NSAID-Related Gastrointestinal Adverse Events in Korean Patients: An Exploratory Genome-Wide Association Study

**DOI:** 10.3390/jcm15187301

**Published:** 2026-09-20

**Authors:** Jun Hyeob Kim, Jin Yeon Gil, Kyung Hyun Min, Hyun Jeong Kim, Soyoun Yang, Sam Yeol Chang, Byung-Ki Cho, Hyoun Ah Kim, Woorim Kim, Jinhyun Kim, In Ah Choi, Nan Song, Ji Min Han, Kyung Eun Lee

**Affiliations:** 1 College of Pharmacy, Chungbuk National University, Cheongju 28160, Republic of Korea; 2 Department of Orthopedic Surgery, Seoul National University Hospital, Seoul National University College of Medicine, Seoul 03080, Republic of Korea; hewl3102@gmail.com; 3 Department of Orthopaedic Surgery, Chungbuk National University Hospital, College of Medicine, Chungbuk National University, Chungbuk 28644, Republic of Korea; titanick25@naver.com; 4 Department of Rheumatology, School of Medicine, Ajou University, Suwon 16499, Republic of Korea; 5 College of Pharmacy, Kangwon National University, Chuncheon 24341, Republic of Korea; 6 Department of Internal Medicine, College of Medicine, Chungnam National University, Daejeon 35015, Republic of Korea; 7 Division of Rheumatology, Department of Internal Medicine, Korea University Guro Hospital, Korea University College of Medicine, Seoul 02841, Republic of Korea

**Keywords:** nonsteroidal anti-inflammatory drugs, gastrointestinal adverse events, pharmacogenomics, genome-wide association study, polygenic risk score, Korean population

## Abstract

**Background/Objectives**: Nonsteroidal anti-inflammatory drugs (NSAIDs) are widely used but can cause clinically significant gastrointestinal adverse events (GI-AEs). However, genetic susceptibility to NSAID-related GI toxicity remains insufficiently characterized in East Asian populations. This exploratory study aimed to identify genetic loci associated with grade ≥ 2 NSAID-related GI-AEs in Korean patients treated with commonly used NSAIDs. **Methods**: We conducted an exploratory genome-wide association study in a multicenter Korean cohort of NSAID-treated patients recruited from four Korean hospitals. A total of 339 patients were included, comprising 28 cases with grade ≥ 2 GI-AEs and 311 controls without documented GI-AEs. Genotyping was performed using the Korea Biobank Array, followed by imputation and quality control. Genome-wide association analysis was conducted under an additive logistic regression model adjusted for age, sex, and the first two principal components, with additional sensitivity analyses accounting for the specific NSAID type, cumulative DDD, and concomitant medications. Polygenic risk score analysis was performed using summary statistics from an arthritis-treated subset of the KoGES Ansan/Ansung cohort, and the selected variant were further evaluated using multivariable Firth penalized logistic regression. The discriminatory performance of clinical, laboratory, and genetic models was assessed using bootstrap internal validation. **Results**: The exploratory GWAS identified 20 suggestive variants associated with NSAID-related GI-AEs at *p* < 1 × 10^−5^, although no variant reached genome-wide significance. The KoGES-derived polygenic risk score was significantly associated with GI-AE status in the NSAID cohort, and 10 suggestive variants overlapped with SNPs included in the best-fit PRS model. rs1925245 maintained a consistent association in multivariable Firth penalized logistic regression. **Conclusions**: These findings suggest that NSAID-related GI-AEs in Korean patients may reflect a multifactorial susceptibility pattern involving both clinical context and polygenic risk. Although exploratory, this study provides candidate genetic signals for future replication and supports the need for larger ancestry-matched pharmacogenomic studies of NSAID-related GI toxicity.

## 1. Introduction

Nonsteroidal anti-inflammatory drugs (NSAIDs) are widely used analgesic and anti-inflammatory agents but remain an important cause of drug-related gastrointestinal (GI) injury [1,2,3]. By inhibiting cyclooxygenase enzymes and reducing the synthesis of protective prostaglandins, NSAIDs can cause dyspepsia, mucosal erosions, peptic ulceration, and gastrointestinal bleeding [2,3,4]. The risk of clinically important upper-GI complications is greater in older patients, those with a history of peptic ulcer disease or GI bleeding, patients with high-dose or prolonged NSAID use, and those using concomitant antiplatelet or anticoagulant therapy [3,4]. NSAID toxicity also extends beyond the stomach and duodenum to the small and large intestine, where it may manifest as increased intestinal permeability, erosions, ulcers, strictures, occult bleeding, and protein-losing enteropathy [3,5].

COX-2-selective inhibitors were developed to preserve anti-inflammatory efficacy while reducing mucosal injury and have demonstrated reduced rates of endoscopically detected ulcers compared with nonselective NSAIDs [6,7,8]. Despite guideline-based risk assessment and gastroprotective strategies, clinically significant gastrointestinal adverse events continue to occur, highlighting the need for improved risk stratification [1,4].

Evidence suggests that individual susceptibility to NSAID-induced GI toxicity may be partly influenced by genetic variation affecting drug metabolism and pharmacologic response [9,10]. However, reported pharmacogenomic associations with NSAID-related GI toxicity have been inconsistent or contradictory, and evidence from East Asian populations remains limited [10,11]. Differences in allele frequencies, linkage disequilibrium patterns, and genetic architecture across ancestry groups may limit the transferability of genetic association findings and polygenic risk scores, supporting the need for ancestry-matched discovery and validation studies in Korean populations [12].

To our knowledge, no prior genome-wide association study (GWAS) has specifically evaluated genetic susceptibility to clinically significant NSAID-related GI-AEs in Korean patients treated with commonly used NSAIDs. Identifying genetic susceptibility markers may contribute to improved risk stratification and the development of personalized gastroprotective strategies. Therefore, we aimed to identify genetic loci associated with grade ≥ 2 NSAID-related GI-AEs in Korean patients treated with commonly used NSAIDs. We further evaluated the robustness of the association signals after adjustment for key clinical covariates and concomitant medications using penalized regression appropriate for sparse outcomes. In addition, we explored shared polygenic susceptibility using an arthritis-treated subset of the KoGES Ansan/Ansung cohort and polygenic risk score analysis.

## 2. Materials and Methods

### 2.1. Study Population and Outcome Definition

Patients treated with NSAIDs, including celecoxib, naproxen, and aceclofenac, were recruited from Chungbuk National University Hospital, Chungnam National University Hospital, Ajou University Hospital, and Seoul National University Hospital. Data collection was conducted from 1 April 2024, to 31 October 2024. These NSAIDs were selected based on their frequent use in routine rheumatology and orthopedic clinical practice at the participating institutions. Cases were defined as patients who developed grade ≥ 2 GI-AEs according to the Common Terminology Criteria for Adverse Events version 5.0, based on the criteria specified for the relevant GI-AE category [13]. Patients with mild GI symptoms classified as grade 1 were excluded to improve phenotypic specificity. Controls were defined as patients who received NSAIDs for a comparable duration without any documented GI-AEs. Sample size was estimated based on a gastrointestinal adverse-events rate of 11.67% reported in a previous clinical study of Korean patients with rheumatoid arthritis receiving celecoxib [14]. Using the formula *n* = Z_1−α/2_^2^p(1 − p)/d^2^, with a 95% confidence level and an absolute precision of ±5%, the minimum required sample size was estimated to be 159 patients.

Clinical data, including demographics, comorbidities, laboratory results, concomitant medications, and NSAID prescription details, including daily dose and duration, were extracted from electronic medical records. Concomitant medications were classified into antidiabetic, antihypertensive, lipid-lowering, anti-arthritic, gastrointestinal, and antithrombotic drug classes. Each class was further grouped by pharmacologic subclass (e.g., biguanides, ARBs, statins, csDMARDs, PPIs, and antiplatelet agents) and standardized to generic ingredient names; the detailed mapping is provided in Supplementary Table S1. Peripheral blood samples were collected, and genomic DNA was extracted using the QIAamp DNA Blood Mini Kit (Qiagen, Hilden, Germany) according to the manufacturer’s protocol.

### 2.2. Genome-Wide Association Analysis

Genotyping was performed using the Korea Biobank Array (Affymetrix, Santa Clara, CA, USA), optimized for the Korean population [15]. Quality control (QC) followed standard procedures. Sample-level QC excluded samples with a Dish Quality Control (DQC) value < 0.82, a genotype call rate < 97%, or heterozygosity rates deviating more than ±2 standard deviations (SD) from the mean. Plate-level QC removed samples from plates with a pass rate ≤ 95% or an average call rate ≤ 99%. Principal component analysis (PCA) was conducted to identify and remove ancestry outliers whose principal component values deviated by more than 3 SD from the mean. After QC, 525,513 single-nucleotide polymorphisms (SNPs) were retained. Genotype imputation was performed using the Korean Imputation Service (KIS) with a population-specific Korean reference panel [16,17]. Post-imputation QC was conducted using PLINK [18], excluding variants with an imputation quality R^2^ < 0.7, a missing genotype rate > 5%, Hardy–Weinberg equilibrium *p* < 1 × 10^−6^, or minor allele frequency (MAF) < 0.01. Consequently, 7,735,084 high-quality variants were available for association analysis. Genome-wide association analysis was performed using PLINK v1.9 [18] under an additive logistic regression model, adjusting for age, sex, PC1, and PC2. PC1 and PC2 represent the first and second principal components derived from principal component analysis of the genotype data and were included as covariates to account for population stratification. To evaluate the potential influence of treatment-related confounding, we performed a sensitivity GWAS under an additive logistic regression model adjusted for age, sex, PC1, PC2, NSAID type, cumulative Defined Daily Dose (DDD), concomitant anti-arthritic medications, and gastrointestinal medications. Cumulative DDD was used to standardize NSAID exposure across different NSAIDs with different prescribed doses. The dependent variable was GI-AE status (case vs. control). Given the limited number of cases (*n* = 28), which constrains statistical power, we adopted a suggestive significance threshold of *p* < 1 × 10^−5^ to identify candidate loci, rather than the conventional genome-wide threshold (*p* < 5 × 10^−8^) [19,20,21]. Manhattan and quantile–quantile (Q–Q) plots were generated using R (v4.4.2) [22]. Independent loci were identified by linkage disequilibrium (LD) clumping using HaploView v4.2 [23], with r^2^ < 0.1 used to define independent signals within a 500-kb window.

In silico functional annotation of the variants was performed using GTEx v8, eQTLGen (https://www.eqtlgen.org/, accessed on 22 April 2026), and Ensembl Variant Effect Predictor (VEP; https://www.ensembl.org/Tools/VEP, accessed on 22 April 2026) to assess significant eQTL associations and predicted functional consequences of the variants [24].

### 2.3. Analysis of Candidate Genes

Candidate CYP2C9 and CYP2C19 alleles were selected based on significant variant annotations reported in ClinPGx for celecoxib, naproxen, and aceclofenac. The corresponding allele-defining variants were identified according to PharmVar and assessed for coverage in the post-quality-control genotyped/imputed dataset. The evaluated alleles included CYP2C9 *1, *2, *3, and *13 and CYP2C19 *1, *9, *14, *16, *18, and *19.

### 2.4. KoGES GWAS and PRS Analysis

Given that most GI-AE cases occurred in patients treated for inflammatory arthritis and frequently received concomitant anti-arthritic medications (Table 1), we additionally leveraged the Korean Genome and Epidemiology Study (KoGES) Ansan/Ansung cohort to perform a complementary GWAS in an arthritis-treated subset (physician-diagnosed arthritis and ≥1 anti-arthritic medication; *n* = 318). KoGES has been described in detail elsewhere [25]. In KoGES, the outcome was GI disease, defined as ≥1 GI diagnosis or ≥1 GI medication, used here as a proxy phenotype for GI adverse events. Using the same QC principles, we conducted an additive logistic regression GWAS for KoGES GI disease and adjusted for age, sex, and PC1–PC2. We then used the KoGES arthritis GWAS summary statistics as the base dataset to construct polygenic risk scores (PRS) in the hospital cohort using PRSice-2 [26]. PRS performance was evaluated by logistic regression of GI-AE status on standardized PRS, adjusting for age, sex, and PC1–PC2; the best-fitting *p*-value threshold was selected according to PRSice-2 output R^2^ and association *p*-value. Among SNPs included in the best-fit KoGES-derived PRS model, we identified NSAID GWAS suggestive variants and tabulated these overlapping PRS-contributing variants (Table 2).

### 2.5. Statistical Analysis

Statistical analyses were performed using SAS version 9.4 (SAS Institute, Cary, NC, USA). Baseline characteristics were compared between cases and controls using Student’s *t*-tests for continuous variables and χ^2^ tests for categorical variables. Associations with GI-AEs were further evaluated using multivariable Firth penalized logistic regression [27]. Odds ratios (ORs) and 95% confidence intervals (CIs) were calculated, with statistical significance defined as *p* < 0.05.

### 2.6. Exploratory ROC Analysis

Exploratory receiver operating characteristic (ROC) analyses were performed to evaluate the apparent discriminatory performance of clinical variables and models augmented with laboratory parameters, concomitant medications, and rs1925245 for GI-AE status. AUCs were estimated across five hierarchical ROC models. Model 1 included age, sex, and BMI. Model 2 further included laboratory parameters, Model 3 concomitant anti-arthritic and gastrointestinal medications, and Model 4 the selected rs1925245. Model 5 included all variables incorporated in Models 1–4. To account for potential optimism in model performance, internal validation was performed using 1000 bootstrap resamples, and optimism-corrected AUCs were calculated by subtracting the estimated optimism from the AUC. Given the limited number of GI-AE cases, these analyses were considered exploratory and were interpreted cautiously rather than as validated prediction performance [28,29].

## 3. Results

To address our study aims, we first describe the clinical characteristics of cases and controls, then report GWAS findings, evaluate exploratory evidence of shared polygenic susceptibility using KoGES, and finally examine robustness in multivariable penalized models.

### 3.1. Baseline Characteristics of the Study Population

To characterize the clinical context of grade ≥ 2 GI-AEs, we compared baseline characteristics and concomitant medication use between cases and controls.

A total of 339 NSAID-treated patients were included, of whom 28 (8.3%) developed grade ≥ 2 gastrointestinal adverse events (GI-AEs) and 311 (91.7%) had no documented events (Table 1). The underlying diagnoses for NSAID treatment were predominantly rheumatoid arthritis (*n* = 182) and ankylosing spondylitis (*n* = 120), with smaller numbers of osteoarthritis (*n* = 18), systemic lupus erythematosus (*n* = 3), and other conditions. The mean duration of NSAID use was 53.7 months.

Compared with controls, cases were more frequently female (78.6% vs. 54.0%, *p* = 0.01), whereas age and BMI did not differ significantly between groups. Smoking status differed between groups (*p* = 0.02), with cases more often being never-smokers. Current smoking was uncommon in both groups (controls 17 vs. cases 2) (Table 1).

Regarding comedications, anti-arthritic medications (50.0% vs. 15.8%, *p* < 0.0001) and gastrointestinal medications (25.0% vs. 7.7%, *p* = 0.002) were more commonly used among cases, and antithrombotic agent use was also higher in cases (7.1% vs. 1.6%, *p* = 0.07). Major comorbidities did not differ significantly between groups, including diabetes mellitus (OR 1.82, 95% CI 0.58–5.66), hypertension (OR 0.47, 95% CI 0.13–1.61), dyslipidemia (OR 2.22, 95% CI 0.84–5.86), and osteoporosis (OR 2.28, 95% CI 0.80–6.50). Compared with controls, cases showed higher white blood cell counts (8.94 vs. 7.65 × 10^3^/µL, *p* = 0.006), bilirubin levels (3.37 vs. 1.74 mg/dL, *p* < 0.0001), and erythrocyte sedimentation rates (89.04 vs. 51.14 mm/h, *p* < 0.0001), along with lower hemoglobin (12.51 vs. 13.29 g/dL, *p* = 0.02) and hematocrit (37.96% vs. 39.91%, *p* = 0.03) (Supplementary Table S2).

### 3.2. Genome-Wide Association Analysis for NSAID-Related Gastrointestinal Adverse Events

To identify genetic susceptibility loci for NSAID-related GI-AEs, we performed a genome-wide association analysis under an additive model.

**Figure 1:**
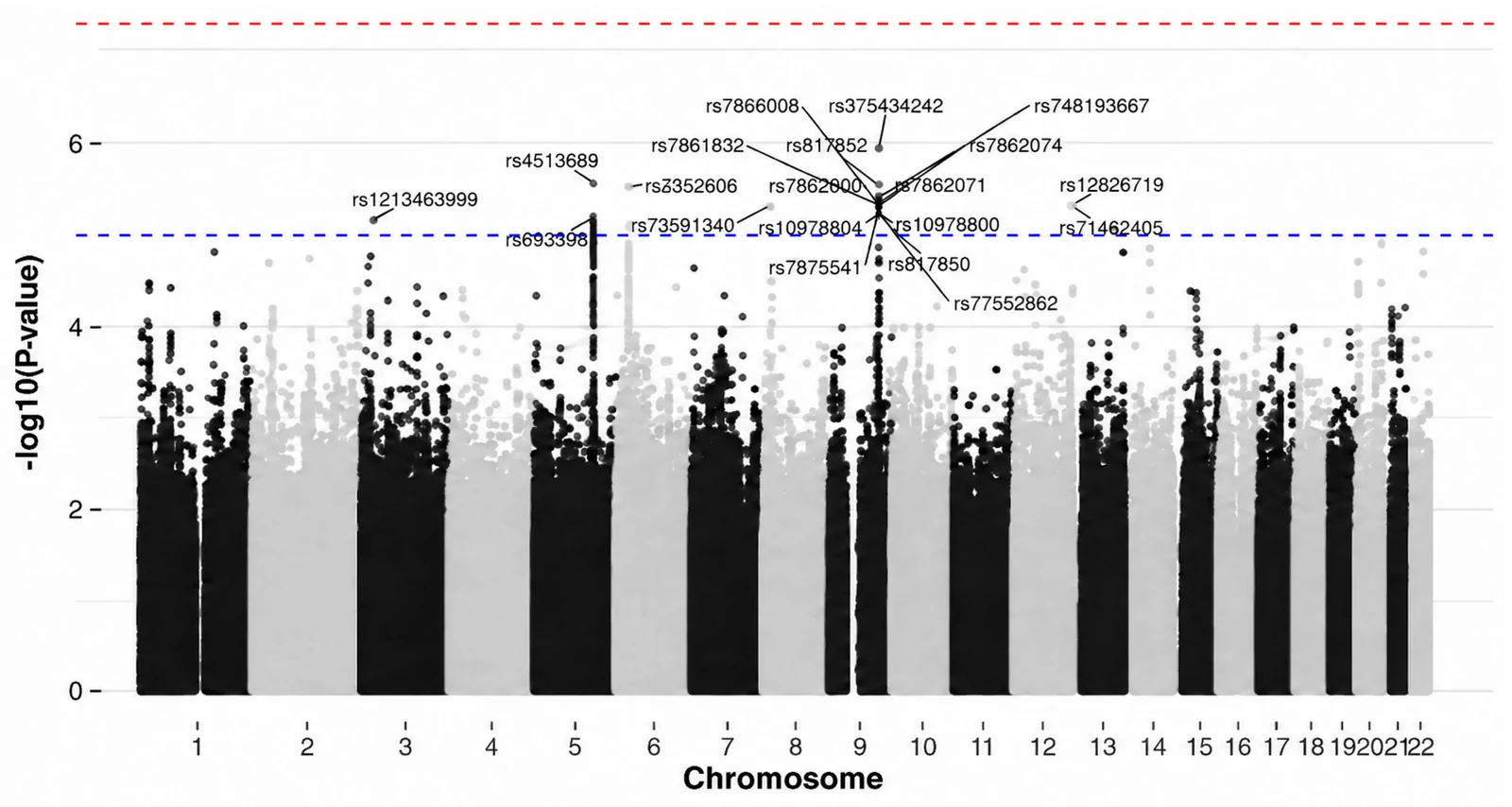
Manhattan plot of the genome-wide association analysis for NSAID-related gastrointestinal adverse events. Genome-wide association results for grade ≥ 2 NSAID-related GI adverse events. Each point represents a single-nucleotide polymorphism (SNP), plotted according to its chromosomal position on the x-axis and –log_10_(*p*) value on the y-axis. The blue dashed line indicates the prespecified suggestive significance threshold (*p* = 1 × 10^−5^), and the red dashed line indicates the conventional genome-wide significance threshold (*p* = 5 × 10^−8^).

After genotyping, quality control, and imputation, 7,735,084 variants were available for association analysis. Genome-wide association analysis comparing cases and controls identified 20 variants surpassing the prespecified suggestive significance threshold (*p* < 1 × 10^−5^) (Supplementary Table S3; Figure 1). Quantile–quantile plots are shown in Supplementary Figure S1. No variant reached the conventional genome-wide significance threshold (*p* < 5 × 10^−8^). The smallest *p*-value observed was 4.45 × 10^−7^, and suggestive signals were distributed across multiple chromosomes (Supplementary Table S3).

In the sensitivity GWAS, adjustment was performed for NSAID type, cumulative Defined Daily Dose (DDD), concomitant anti-arthritic medications, and gastrointestinal medications. Several suggestive association signals remained, although no variant reached genome-wide significance. rs74475327 and rs1925245 were consistently identified in both the primary and additionally adjusted GWAS analyses (Supplementary Table S4 and Supplementary Figure S2).

### 3.3. Association Results for Candidate Genes

Among the CYP2C9 and CYP2C19 variants evaluated, rs1057910 in CYP2C9 and rs3758581 in CYP2C19 were present in the post-quality-control genotyped/imputed dataset, whereas the remaining candidate variants were not present after quality control. The ORs for GI-AE occurrence were 3.13 (95% CI, 0.73–13.30) for rs1057910 and 1.75 (95% CI, 0.44–7.01) for rs3758581. Neither variant showed a statistically significant association with GI-AE occurrence (Supplementary Table S5).

### 3.4. KoGES Arthritis GWAS Comparison and Polygenic Risk Score Analysis

To assess shared polygenic susceptibility in an arthritis-treatment context and evaluate polygenic transferability, we conducted a complementary GWAS among arthritis-treated participants in the KoGES Ansan/Ansung cohort (participants reporting both an arthritis diagnosis and anti-arthritic medication use; *n* = 318). Within this subset, 56 participants met the KoGES GI disease definition, whereas 262 did not. The mean age was 60.67 ± 8.49 years in cases and 62.51 ± 8.89 years in controls, and females accounted for 45/56 (80.36%) of cases and 194/262 (74.05%) of controls.

Using the KoGES arthritis GWAS summary statistics as the base dataset, we constructed polygenic risk scores (PRS) in the hospital cohort using PRSice-2. The PRS was significantly associated with GI-AE status (PRSice-2 output: PRS.R^2^ = 0.0366367; *p* = 0.0210122; best-fit *p*-value threshold = 0.00840005; number of SNPs included = 4956) after adjustment for age, sex, and PC1–PC2. Among the SNPs included in the best-fit KoGES-derived PRS model, 10 were NSAID GWAS suggestive variants (Table 2), providing exploratory evidence of shared polygenic susceptibility in an arthritis-treatment context. Manhattan and quantile–quantile plots for the KoGES GWAS are provided in Supplementary Figures S3 and S4.

In silico functional annotation using GTEx v8 identified gastrointestinal tissue-specific eQTL associations for several variants included in Table 2 (Supplementary Table S6). rs4513689 was associated with HINT1 expression in stomach tissue, whereas rs1925245 was associated with PLCE1 expression in transverse colon tissue. rs7259854 showed multiple eQTL associations in stomach, terminal ileum, sigmoid colon, and transverse colon tissues. No significant gastrointestinal tissue eQTL associations were identified for the remaining evaluated variants.

### 3.5. Multivariable Models

To evaluate whether the observed associations were robust to adjustment for clinical covariates, we performed multivariable Firth penalized logistic regression using hierarchical models (Table 3). Among the 20 suggestive variants identified in the primary GWAS, rs1925245 remained suggestive in the sensitivity GWAS and was also included in the KoGES-derived PRS model. Because rs1925245 showed consistent support across the primary GWAS, sensitivity GWAS, and complementary KoGES analysis, it was selected for subsequent multivariable analysis together with relevant clinical covariates. In Model 2, higher albumin levels were associated with a lower risk of GI-AEs (OR 0.22, 95% CI 0.05–0.57), whereas higher white blood cell counts were associated with an increased risk (OR 1.27, 95% CI 1.02–1.63). In Model 3, female sex was associated with an increased risk of GI-AEs (OR 3.09, 95% CI 1.17–9.44), and concomitant anti-arthritic medication use was also associated with an increased risk (OR 5.42, 95% CI 2.14–13.91). In Model 4, rs1925245 was associated with a lower risk of GI-AEs (OR 0.04, 95% CI 0.01–0.17). In the fully adjusted Model 5, rs1925245 remained associated with a lower risk of GI-AEs (OR 0.03, 95% CI 0.003–0.24), while concomitant anti-arthritic medication use remained associated with an increased risk (OR 7.34, 95% CI 2.04–31.22).

### 3.6. Exploratory Discrimination Analysis 

Exploratory ROC analyses were performed using five models. Model 1, including age, sex, and BMI, showed an AUC of 0.665 and an optimism-corrected AUC of 0.626. Compared with Model 1, the optimism-corrected AUC increased by 0.231 with the addition of laboratory parameters (Model 2), by 0.108 with concomitant medications (Model 3), and by 0.106 with rs1925245 (Model 4). The fully adjusted Model 5 showed the highest optimism-corrected AUC (0.914), representing an increase of 0.288 compared with Model 1 (Supplementary Table S7 and Supplementary Figure S5).

## 4. Discussion

In this multicenter Korean NSAID-exposed cohort (28 grade ≥ 2 GI-AE cases; 311 controls), we aimed to identify genetic susceptibility loci for clinically significant NSAID-related GI-AEs and to evaluate robustness and explore shared polygenic susceptibility using a complementary KoGES arthritis-treated GWAS, and PRS analysis. We observed that clinically significant GI-AEs clustered in an inflammatory arthritis treatment context with frequent concomitant anti-arthritic and GI medication use, and the exploratory GWAS identified 20 suggestive variants (*p* < 1 × 10^−5^) without genome-wide significant hits. Among these, rs1925245 remained suggestive in the sensitivity GWAS after adjustment for NSAID type, cumulative DDD, and concomitant medications and was also included in the KoGES-derived PRS model. The consistent observation of rs1925245 across the primary GWAS, sensitivity GWAS, and complementary PRS analysis suggests that this variant may represent a candidate locus for NSAID-related GI-AEs. These findings may provide population-specific insight into interindividual susceptibility to NSAID-related GI-AEs.

From a pharmacogenetic perspective, genetic factors may contribute to interindividual variability in susceptibility to NSAID-related GI toxicity [10]. Previous studies have identified genetic associations with NSAID-related gastrointestinal injury, including variants in *CYP2C9* and *CYP2C8* associated with NSAID-related gastrointestinal bleeding [9,30]. An intronic variant in *EYA1* was associated with aspirin-induced peptic ulceration [19] and variants in *ALOX15* and *PTGER4* were associated with gastrointestinal toxicity among celecoxib-treated patients [31]. In Korean individuals, *IL1B* variants have been associated with aspirin-induced peptic ulcer, while genetic variation in *PTGS2* has been reported to influence the pharmacodynamic response to celecoxib [32,33]. However, genetic susceptibility to clinically significant GI-AEs during long-term NSAID treatment has not been well characterized in Korean populations.

Our findings are consistent with established mechanisms of NSAID-induced GI toxicity, in which COX inhibition reduces protective prostaglandin synthesis and compromises gastrointestinal mucosal integrity [2,3,34]. Among the suggestive loci identified in our study, rs1925245 is located in PLCE1, which encodes phospholipase C epsilon 1, a protein involved in intracellular signaling. In our functional annotation, rs1925245 was also associated with PLCE1 expression in transverse colon tissue (Supplementary Table S6). Experimental evidence suggests that PLCE1 can modulate PKC and NF-κB signaling [35] and NF-κB signaling has been implicated in indomethacin-induced gastric mucosal injury [36]. In addition, NF-κB has been shown to regulate COX-2 expression in human gastric cells [37]. These observations provide a potential biological link between PLCE1 and signaling pathways relevant to NSAID-induced gastrointestinal injury. However, the functional effect of rs1925245 on PLCE1 activity, COX signaling, or gastrointestinal mucosal integrity remains to be established.

Key strengths of this study include a clinically anchored phenotype focusing on grade ≥ 2 GI-AEs, multicenter recruitment with detailed clinical and medication information, Korean-optimized genotyping with population-specific imputation, and complementary analyses using KoGES and polygenic risk score analyses to explore shared polygenic susceptibility [15,16,17,25,26]. In addition, Firth penalized logistic regression was used to reduce small-sample bias and separation associated with sparse outcomes [27].

Several limitations should be acknowledged. First, the small number of GI-AE cases substantially limited statistical power and the precision of effect estimates, and no variant reached genome-wide significance [38]. Second, the KoGES analysis provides complementary evidence but cannot be considered direct replication because the proxy GI phenotype used in KoGES differed from the clinically adjudicated grade ≥ 2 GI-AE phenotype used in the hospital cohort. Third, although treatment-related factors were additionally adjusted for in the sensitivity analyses, residual confounding related to heterogeneous NSAID exposure and concomitant medications may remain. Finally, the ROC/AUC analyses were internally validated but remain exploratory given the limited sample size and should not be interpreted as evidence of validated predictive performance.

## 5. Conclusions

This exploratory genome-wide association study suggests that clinically significant NSAID-related gastrointestinal adverse events in Korean patients may reflect a multifactorial susceptibility pattern involving both clinical context and polygenic risk. Although the identified loci and KoGES-derived PRS findings support a potential genetic contribution, these results should be interpreted as hypothesis-generating and require replication in larger ancestry-matched cohorts with harmonized phenotype definitions.

## Figures and Tables

**Table 1:** Baseline clinical and laboratory characteristics of NSAID-treated patients with and without grade ≥ 2 gastrointestinal adverse events.

		Control (*n* = 311)	Case (*n* = 28)	*p*-Value	OR (95% CI)
Age, years (mean ± SD)		51.00 ± 14.51	56.10 ± 13.52	0.07	1.02 (0.99–1.06)
Sex	Male (ref.)	143 (45.98)	6 (21.43)		
	Female	168 (54.02)	22 (78.57)	0.01	3.12 (1.23–7.90)
Weight, kg		64.87 ± 12.95	59.88 ± 12.09	0.06	0.96 (0.93–1.00)
BMI, kg/m^2^		23.90 ± 3.51	24.12 ± 3.98	0.77	1.01 (0.90–1.14)
Alcohol		234 (75.48)	25 (89.29)	0.09	
Smoking	Never	226 (72.58)	26 (92.86)	0.02	
	Former	68 (21.94)	0 (0.00)		
	Current	17 (5.48)	2 (7.14)		
NSAID type	Celecoxib (ref.)	149 (47.91)	18 (64.29)	0.14	
	Naproxen	82 (26.37)	3 (10.71)		0.30 (0.08–1.05)
	Aceclofenac	80 (25.72)	7 (25.00)		0.72 (0.29–1.81)
Defined Daily Dose		27.20 (23.64)	22.33 (26.85)		0.99 (0.97–1.01)
Comorbidity					
Diabetes mellitus		26 (8.36)	4 (14.29)	0.29	1.82 (0.58–5.66)
Hypertension		63 (20.26)	3 (10.71)	0.22	0.47 (0.13–1.61)
Dyslipidemia		34 (10.93)	6 (21.43)	0.09	2.22 (0.84–5.86)
Osteoporosis		27 (8.68)	5 (17.86)	0.11	2.28 (0.80–6.50)
Comedication					
Antidiabetic medications		10 (3.22)	1 (3.57)	0.91	1.11 (0.13–9.04)
Antihypertensive medications		40 (12.86)	1 (3.57)	0.14	0.25 (0.03–1.89)
Lipid-lowering agents		21 (6.75)	2 (7.14)	0.93	1.06 (0.23–4.78)
Anti-arthritic medications		49 (15.76)	14 (50.00)	<0.01	5.34 (2.40–11.91)
Gastrointestinal medications		24 (7.72)	7 (25.00)	<0.01	3.98 (1.53–10.32)
Antithrombotic agents		5 (1.61)	2 (7.14)	0.07	4.70 (0.87–25.46)

Note: Values are mean ± SD or *n* (%). For sex and NSAID type, ORs are relative to the reference categories (male and celecoxib, respectively).

**Table 2:** NSAID GWAS suggestive variants included in the best-fit KoGES-derived PRS model.

SNP	CHR	BP	Gene	Consequence	Ref	Alt	OR
rs12729383	1	86509456	COL24A1	Intron Variant	G	A	9.17 (3.40–24.68)
rs4513689	5	130368978	LOC105379172	Intron Variant	T	C	3.89 (2.15–7.05)
rs73591340	8	18965159	LOC105379301	Intron Variant	T	G	4.01 (2.23–7.21)
rs817852	9	110106222	-	-	C	T	0.23 (0.12–0.43)
rs1925245	10	95785262	PLCE1	Intron Variant	T	G	0.08 (0.03–0.26)
rs77396079	13	90541088	-	-	A	G	7.73 (3.25–18.38)
rs78504122	16	22035166	MOSMO	Intron Variant	C	T	9.34 (3.62–24.11)
rs7259854	19	36685753	ZNF565	Intron Variant	A	G	10.03 (3.46–29.02)
rs3026502	20	57946750	-	-	G	A	7.02 (3.03–16.23)
rs77819704	21	31329573	-	-	G	A	8.54 (3.11–23.48)

SNPs listed are NSAID GWAS suggestive variants that were also included in the best-fit KoGES-derived PRS model. CHR: chromosome; BP: base-pair position. Gene denotes the nearest annotated gene and consequence indicates the predicted functional annotation. Ref and Alt indicate the reference and alternative alleles, respectively. ORs are reported per Alt allele under an additive model as estimated in the hospital cohort post-GWAS regression framework described in Methods. “-” indicates no gene annotation available at the locus.

**Table 3:** Firth penalized logistic regression models for grade ≥ 2 gastrointestinal adverse events.

Variable	Model 1	Model 2	Model 3	Model 4	Model 5
Age	1.02 (0.99–1.05)	1.00 (0.97–1.03)	1.01 (0.98–1.05)	1.02 (0.99–1.05)	1.01 (0.97–1.05)
Sex (1 vs. 0)	2.49 (0.97–7.47)	2.85 (0.78–11.87)	3.09 (1.17–9.44)	3.64 (1.26–13.15)	3.42 (0.80–18.85)
BMI	1.04 (0.92–1.16)	1.06 (0.91–1.24)	1.05 (0.93–1.19)	1.00 (0.87–1.13)	1.02 (0.85–1.20)
Hb	-	1.03 (0.73–1.49)	-	-	0.84 (0.56–1.26)
Neutrophil	-	1.00 (0.95–1.05)	-	-	1.00 (0.94–1.06)
Albumin	-	0.22 (0.05–0.57)	-	-	0.92 (0.01–1.14)
Bilirubin	-	0.65 (0.25–1.54)	-	-	2.14 (0.19–4.23)
WBC	-	1.27 (1.02–1.63)	-	-	1.26 (0.96–1.68)
ESR	-	0.99 (0.98–1.00)	-	-	1.00 (0.98–1.01)
Anti-arthritic medications	-	-	5.42 (2.14–13.91)	-	7.34 (2.04–31.22)
Gastrointestinal medications	-	-	1.34 (0.37–4.22)	-	2.41 (0.43–12.94)
rs1925245	-	-	-	0.04 (0.01–0.17)	0.03 (0.003–0.24)

Values are presented as odds ratios (ORs) with 95% confidence intervals (CIs). Model 1 adjusted for age, sex, and BMI. Model 2 additionally included laboratory parameters (hemoglobin, neutrophil count, albumin, bilirubin, white blood cell count, and erythrocyte sedimentation rate). Model 3 additionally included concomitant anti-arthritic and gastrointestinal medications. Model 4 included age, sex, BMI, and rs1925245. Model 5 included age, sex, BMI, laboratory parameters, concomitant anti-arthritic and gastrointestinal medications, and rs1925245. A hyphen (-) indicates that the variable was not included in the model. BMI: body mass index; Hb: hemoglobin; WBC: white blood cell count; ESR: erythrocyte sedimentation rate.

## Data Availability

The data presented in this study are not publicly available due to privacy and ethical restrictions.

## Supplementary Materials

The following supporting information can be downloaded at: https://www.mdpi.com/article/10.3390/jcm15187301/s1, Table S1: Classification of concomitant medications; Table S2: Laboratory findings in NSAID-treated patients with and without grade ≥ 2 GI-AEs; Table S3: Suggestive variants associated with NSAID-related grade ≥ 2 gastrointestinal adverse events in the NSAID GWAS (*p* < 1 × 10^−5^); Table S4: Suggestive variants identified in the sensitivity GWAS for NSAID-related grade ≥ 2 gastrointestinal adverse events (*p* < 1 × 10^−5^); Table S5: Coverage of selected CYP2C9 and CYP2C19 variants in the post-quality-control genotyped/imputed dataset; Table S6: Functional annotation of variants included in Table 2 using GTEx v8, eQTLGen, and Ensembl VEP; Table S7: AUCs and optimism-corrected AUCs from bootstrap internal validation of multivariable models; Figure S1: Quantile–quantile plot of genome-wide association *p* values for NSAID-related grade ≥ 2 GI-AEs; Figure S2: Manhattan plot of the sensitivity GWAS for NSAID-related gastrointestinal adverse events; Figure S3: Manhattan plot of the KoGES arthritis-treated GWAS for GI disease; Figure S4: Quantile–quantile plot of the KoGES arthritis-treated GWAS for GI disease; Figure S5: ROC curves of the five exploratory discrimination models.
