# Supplementary material for: Genetic Susceptibility to NSAID-Related Gastrointestinal Adverse Events in Korean Patients: An Exploratory Genome-Wide Association Study
Source: J Clin Med. 2026 Sep 20;15(18):7301. doi: 10.3390/jcm15187301 (PMC13607881; doi:10.3390/jcm15187301)
Supplement: Supplementary file 1 [file jcm-15-07301-s001.zip › jcm-4466952-supplementary.pdf]

Supplementary Table S1. Classification of concomitant medications

Drug class	Subclass	Generic ingredients
Antidiabetic medications	Biguanides	metformin
	Sulfonylureas	glimepiride
	DPP-4 inhibitors	sitagliptin; teneligliptin
	DPP-4 inhibitor + metformin (FDC)	vildagliptin/metformin; linaglip tin/metformin
Antihypertensive medications	Calcium channel blockers (CCBs)	amlodipine; diltiazem
	Angiotensin II receptor block ers (ARBs)	candesartan; losartan; valsar tan; telmisartan; olmesartan
	Beta-blockers	nebivolol; carvedilol
	ARB + CCB (FDC)	telmisartan/amlodipine
Lipid-lowering agents	Statins (HMG-CoA reductas e inhibitors)	atorvastatin; rosuvastatin; pit avastatin
	Cholesterol absorption inhibi tor	ezetimibe
	Statin + ezetimibe (FDC)	rosuvastatin/ezetimibe
	Fibrates (PPAR-alpha agoni sts)	fenofibrate
Anti-arthritic medications	csDMARDs	hydroxychloroquine; leflunom ide; methotrexate; sulfasalaz ine
	bDMARDs (TNF inhibitors)	adalimumab; infliximab; goli mumab
	Glucocorticoids	prednisolone; methylpredniso lone; deflazacort; triamcinolo ne
	Other arthritis-related agents	colchicine; diacerein
Gastrointestinal medications	Proton pump inhibitors (PPI s)	omeprazole; lansoprazole; p antoprazole; rabeprazole
	Potassium-competitive acid blocker (P-CAB)	tegoprazan
	H2 receptor antagonists (H2 blockers)	famotidine; lafutidine; nizatidi ne; ranitidine
	Mucosal protective / anti-ulc er agents	rebamipide; teprenone; sucr alfate
Antithrombotic agents	Antiplatelet agents	aspirin; clopidogrel
	Anticoagulants (vitamin K a ntagonist)	warfarin

Abbreviations: ARB, angiotensin II receptor blocker; CCB, calcium channel blocker; csDMARD, conventional synthetic DMARD; bDMARD, biologic DMARD; FDC, fixed-dose combination; P-CAB, potassium-competitive acid blocker; PPI, proton pump inhibitor.

Supplementary Table S2. Laboratory findings in NSAID-treated patients with and without grade ≥2 GI-AEs

	Control (n=311)<br>mean ± SD	Case (n=28)<br>mean ± SD	P-value
White Blood Cell, 10^3/μL	7.65 ± 2.23	8.94 ± 3.16	0.006
Red Blood Cell, 10^6/μL	4.38 ± 0.48	4.31 ± 0.49	0.47
Hemoglobin, g/dL	13.29 ± 1.67	12.51 ± 1.50	0.02
Hematocrit, %	39.91 ± 4.64	37.96 ± 4.15	0.03
Platelet, 10^3/μL	285.2 ± 80.20	312.9 ± 97.69	0.09
Mean Corpuscular Hemoglobin, pg	30.34 ± 2.40	29.08 ± 2.84	0.01
Mean Corpuscular Hemoglobin Concentration, g/dL	33.27 ± 1.06	32.95 ± 1.01	0.13
Mean Corpuscular Volume, fL	91.12 ± 5.79	88.20 ± 7.02	0.01
Neutrophil, %	62.10 ± 11.46	66.75 ± 11.03	0.04
Lymphocyte, %	28.11 ± 9.85	24.08 ± 10.20	0.04
Monocyte, %	7.35 ± 2.52	6.98 ± 2.23	0.46
Eosinophil, %	1.81 ± 1.68	1.58 ± 1.34	0.48
Basophil, %	0.59 ± 0.32	0.60 ± 0.26	0.87
Glucose, mg/dL	106.1 ± 27.19	110.6 ± 24.38	0.41
Albumin, g/dL	2.84 ± 2.09	0.68 ± 0.19	<0.0001
Bilirubin, mg/dL	1.74 ± 1.52	3.37 ± 0.51	<0.0001
Erythrocyte Sedimentation Rate, mm/hr	51.14 ± 44.65	89.04 ± 59.52	<0.0001
C-reactive Protein, mg/dL	0.84 ± 2.10	0.46 ± 0.14	0.37

Values are presented as mean ± SD. P-values were calculated using two-sided Student's t-tests comparing cases and controls. Units are shown in the table; differential counts (neutrophil, lymphocyte, monocyte, eosinophil, basophil) are presented as percentages. Missing values, if any, were excluded pairwise for each laboratory variable. GI-AE: gastrointestinal adverse event.

Supplementary Figure S1. Quantile–Quantile plot of genome-wide association P values for NSAID-related grade ≥2 GI-AEs

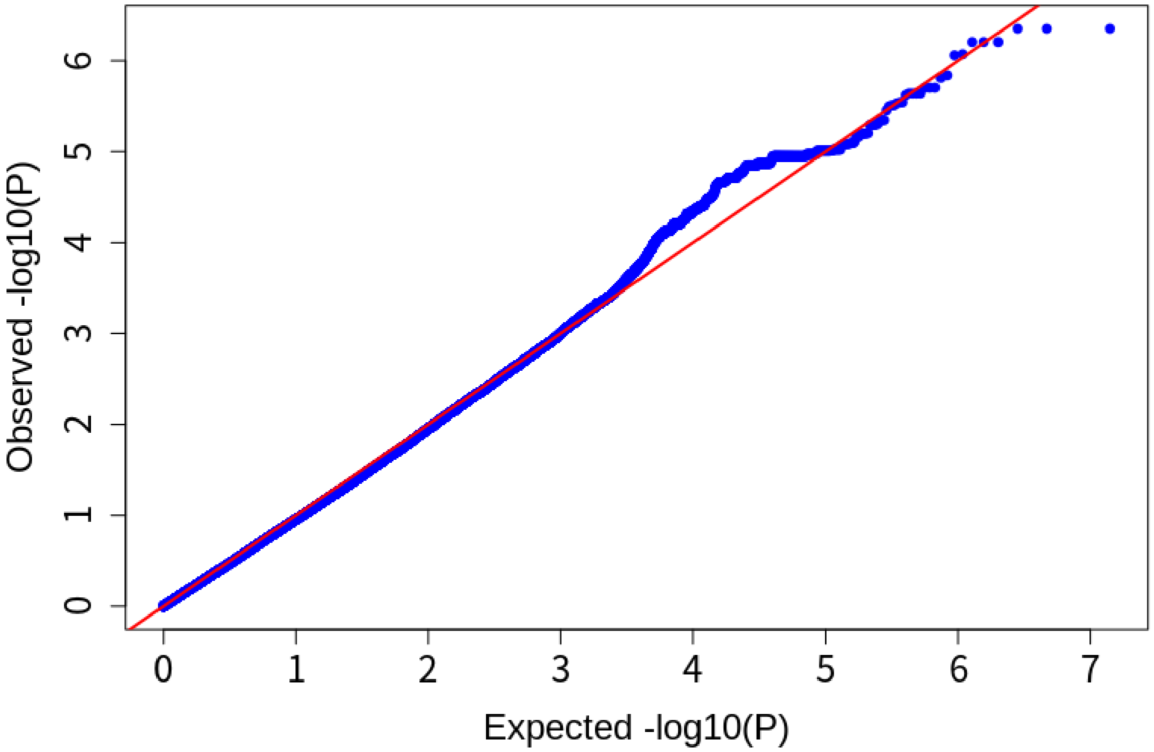

Quantile–quantile (Q–Q) plot comparing the observed $–\log_{10}$(P) values from the GWAS of grade ≥2 NSAID-related GI adverse events with those expected under the null hypothesis. Deviation from the diagonal line in the upper tail reflects SNPs with stronger-than-expected associations.

Supplementary Table S3. Suggestive variants associated with NSAID-related grade ≥2 gastrointestinal adverse events in the NSAID GWAS ($P < 1×10^{-5}$)

SNP	CHR	BP	Gene	Consequence	Ref	Alt	P-value
rs12729383	1	86509456	COL24A1	Intron Variant	G	A	8.22×10^{-6}
rs74475327	3	22071813	ZNF385D	Intron Variant	C	T	8.72×10^{-7}
rs7619163	3	29450797	RBMS3	Intron Variant	C	A	4.45×10^{-7}
rs140789738	4	148033901			T	C	6.47×10^{-6}
rs9292214	5	59112984	PDE4D	Intron Variant	C	T	8.27×10^{-6}
rs4513689	5	130368978	LOC105379172	Intron Variant	T	C	3.5×10^{-6}
rs73591340	8	18965159	LOC105379301	Intron Variant	T	G	4.51×10^{-6}
rs817852	9	110106222			C	T	3.18×10^{-6}
rs142858292	9	139585717	AGPAT2	Intron Variant	T	C	8.55×10^{-6}
rs1925245	10	95785262	PLCE1	Intron Variant	T	G	6.86×10^{-6}
rs71462405	12	128385134	LINC00508	Intron Variant	C	T	4.5×10^{-6}
rs77396079	13	90541088			A	G	8.09×10^{-6}
rs9555366	13	108158536	NALF1	Intron Variant	A	G	3.1×10^{-6}
rs139749713	14	68639372	RAD51B	Intron Variant	C	T	2.39×10^{-6}
rs78504122	16	22035166	MOSMO	Intron Variant	C	T	2.94×10^{-6}
rs147994453	18	41879746			G	A	9.8×10^{-6}
rs7259854	19	36685753	ZNF565	Intron Variant	A	G	3.09×10^{-6}
rs3026502	20	57946750			G	A	9.75×10^{-6}
rs7273513	20	62817277	MYT1	Intron Variant	G	A	2.29×10^{-6}

rs77819704	21	31329573		G	A	9.79×10^{-6}

List of single nucleotide polymorphisms (SNPs) reaching the prespecified suggestive significance threshold ($P < 1\times10^{-5}$) for association with NSAID-related grade ≥2 GI adverse events in the NSAID cohort. The table includes chromosome (CHR), base-pair position (BP), nearest annotated gene, predicted functional consequence, reference (Ref) and alternate (Alt) alleles, and association P-values. SNP: single nucleotide polymorphism; CHR: chromosome; BP: base-pair position.

Supplementary Table S4. Suggestive variants identified in the sensitivity GWAS for NSAID-related grade ≥2 gastrointestinal adverse events ($P < 1×10^{-5}$)

SNP	CHR	BP	Gene	Consequence	Ref	Alt	P-value
rs73016440	1	163762653	LOC124904447	Intron Variant	T	A	$3.28 × 10^{-6}$
rs5746040	1	12255748	TNFRSF1B	Intron Variant	G	C	$5.76 × 10^{-6}$
rs74475327	3	22071813	ZNF385D	Intron Variant	C	T	$6.32 × 10^{-6}$
rs143558088	5	6812323	LINC02236	Intron Variant	C	T	$6.32 × 10^{-6}$
rs1356253	7	80084626	–	–	G	A	$8.39 × 10^{-6}$
rs9408796	9	9848848	PTPRD	Intron Variant	T	C	$6.25 × 10^{-6}$
rs183961034	10	70843518	–	–	T	A	$7.68 × 10^{-6}$
rs1925245	10	95785262	PLCE1	Intron Variant	T	G	$9.38 × 10^{-6}$

List of single nucleotide polymorphisms (SNPs) labeled in the sensitivity GWAS Manhattan plot that reached the prespecified suggestive significance threshold ($P < 1×10^{-5}$). The sensitivity GWAS was adjusted for age, sex, PC1, PC2, NSAID type, Defined Daily Dose, concomitant anti-arthritic medications, and gastrointestinal medications. The table includes chromosome (CHR), base-pair position (BP), nearest annotated gene, predicted functional consequence, reference (Ref) and alternate (Alt) alleles, and association P-values. SNP: single nucleotide polymorphism; CHR: chromosome; BP: base-pair position.

Supplementary Figure S2. Manhattan plot of the sensitivity GWAS for NSAID-related gastrointestinal adverse events

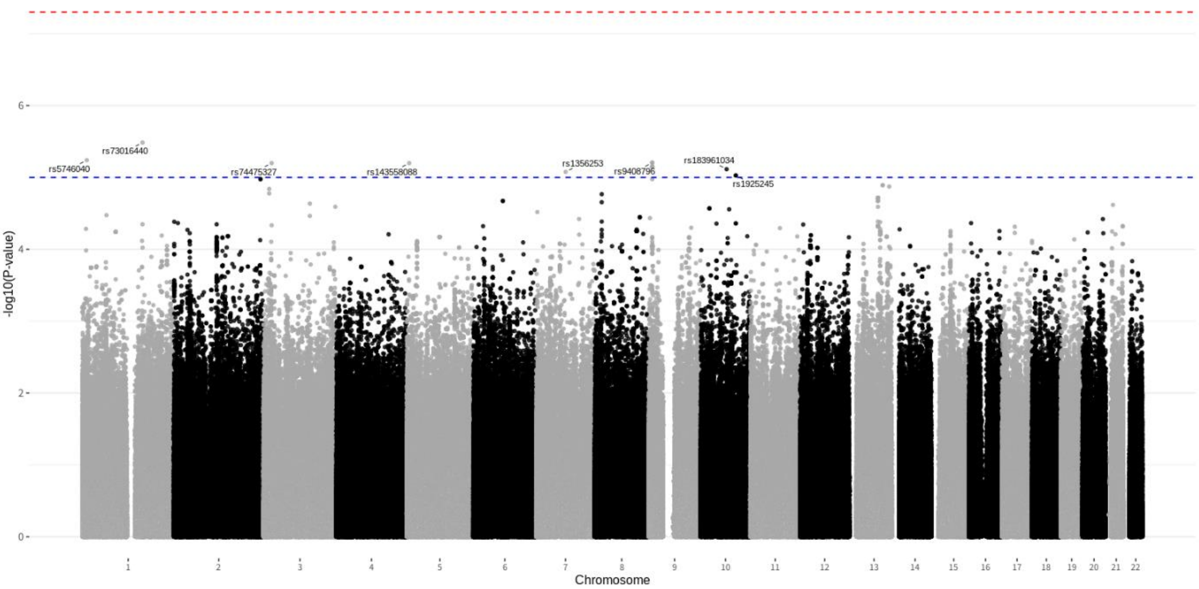

Manhattan plot of the sensitivity GWAS additionally adjusted for age, sex, PC1, PC2, NSAID type, Defined Daily Dose, concomitant anti-arthritic medications, and gastrointestinal medications. Each point represents a single nucleotide polymorphism (SNP), plotted according to its chromosomal position on the x-axis and $-\log_{10}(P)$ value on the y-axis. The blue dashed line indicates the suggestive significance threshold ($P = 1 \times 10^{-5}$), and the red dashed line indicates the conventional genome-wide significance threshold ($P = 5 \times 10^{-8}$).

Supplementary Table S5. Coverage of selected CYP2C9 and CYP2C19 variants in the post-quality-control genotyped/imputed dataset

Gene	Variant (rsID)	Post-QC coverage status	OR (95% CI)
CYP2C9	rs1799853	Not present after QC	
	rs1057910	Present	3.13 (0.73-13.30)
	rs72558187	Not present after QC	
CYP2C19	rs3758581	Present	1.75 (0.44-7.01)
	rs17884712	Not present after QC	
	rs55752064	Not present after QC	
	rs192154563	Not present after QC	
	rs138142612	Not present after QC	
	rs1564657013	Not present after QC	

Candidate pharmacogenes were selected based on ClinPGx Variant Annotations for celecoxib, naproxen, and aceclofenac, and relevant variants were identified using PharmVar. Coverage was assessed in the post-quality-control genotyped/imputed dataset. “Present” indicates that the variant was retained in the final post-QC dataset; “Not present after QC” indicates that the variant was not retained in the final post-QC dataset and does not necessarily indicate absence from the pre-QC imputed data.

Supplementary Figure S3. Manhattan plot of the KoGES arthritis-treated GWAS for GI disease

Manhattan plot of genome-wide association results for KoGES GI disease (proxy phenotype) within the arthritis-treated subset. The horizontal dashed line indicates the prespecified suggestive significance threshold (P = 1×10⁻⁵).

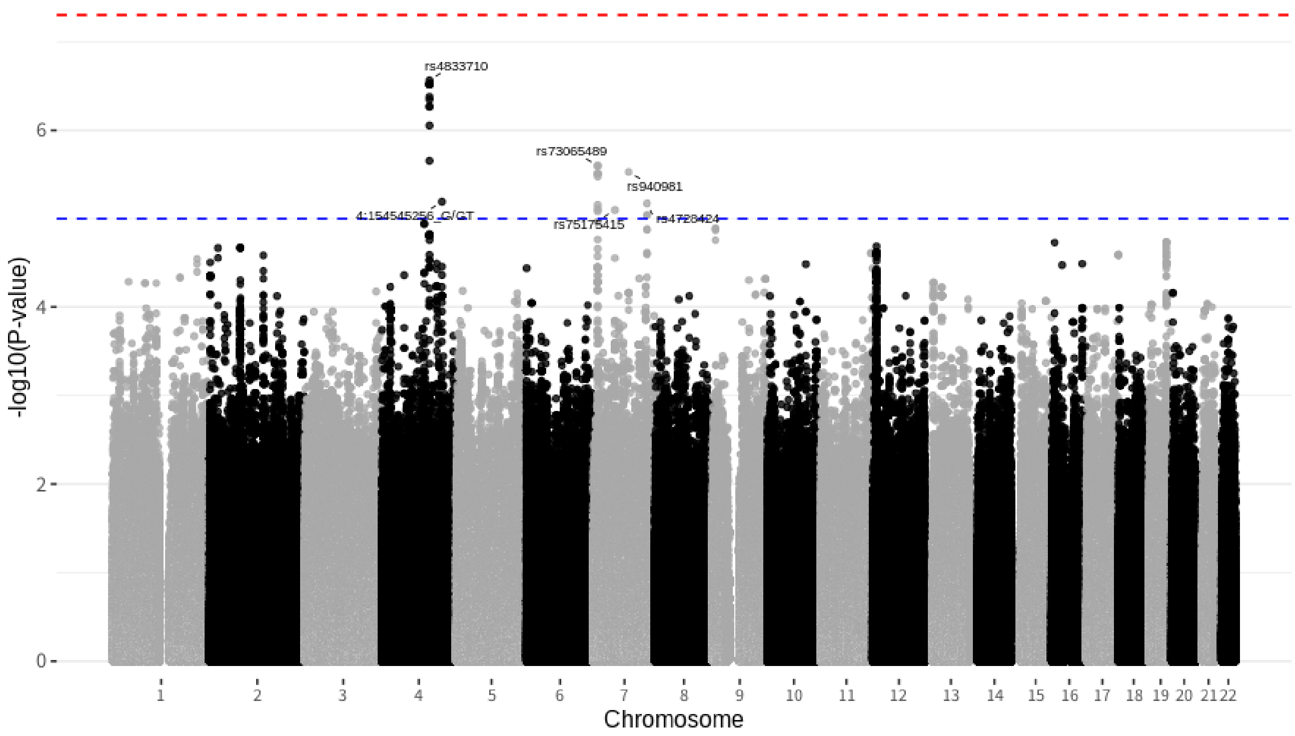

Supplementary Figure S4. Quantile–Quantile plot of the KoGES arthritis-treated GWAS for GI disease

Quantile–quantile (Q–Q) plot comparing observed $–\log_{10}$(P) values from the KoGES arthritis-treated GWAS with those expected under the null hypothesis. Deviation from the diagonal line in the upper tail reflects SNPs with stronger-than-expected associations.

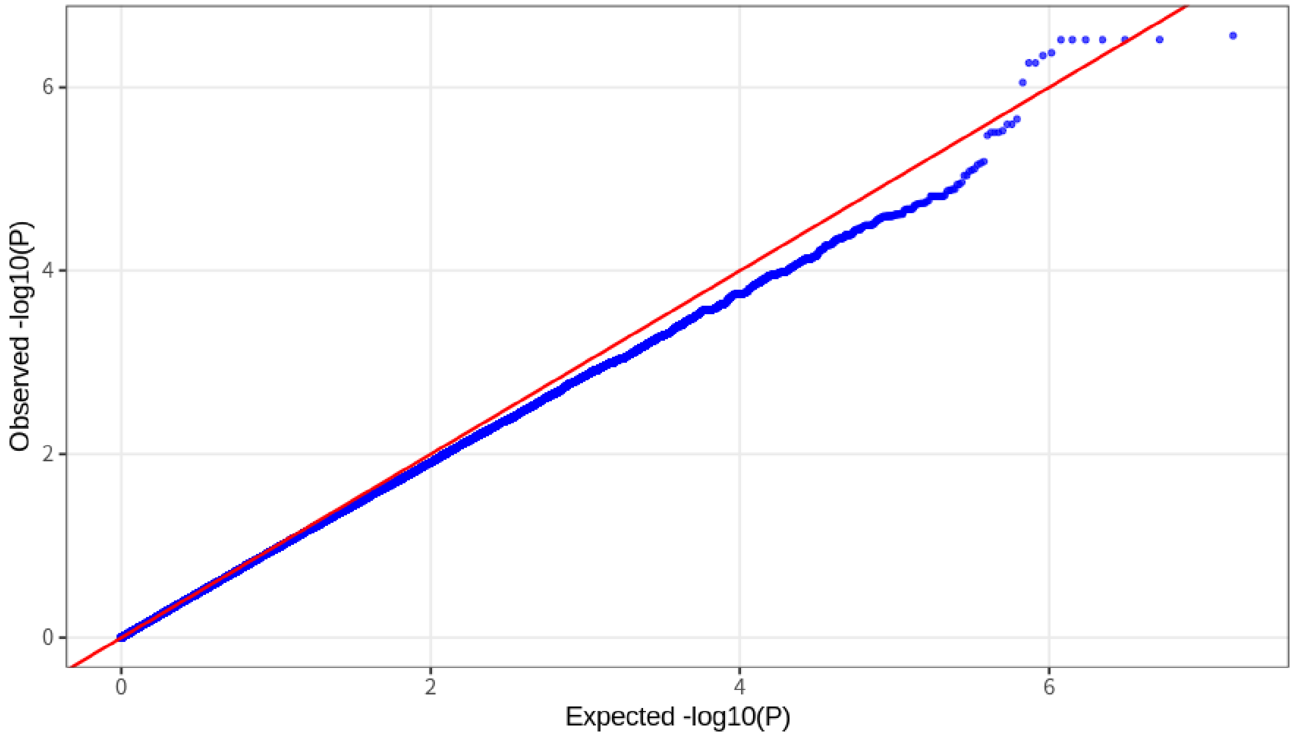

Supplementary Table S6. Functional annotation of variants included in Table 2 using GTEx v8, eQTLGen, and Ensembl VEP

SNP	Source	Tissue	Target gene	Effect	P-value	Functional annotation
**rs12729383**	GTEx v8	Queried GI tissues	No significant eQTL identified	–	–	
**rs12729383**	eQTLGen Phase I	Blood	ZNHIT6	Z 8.5981	8.11×10^{-18}	
**rs12729383**	Ensembl VEP	–	–	–	–	Gene: COL24A1; most severe consequence: intron_variant; biotype: nonsense_mediated_decay; protein_coding; transcript consequence: NMD_transcript_variant; intron_variant
**rs4513689**	GTEx v8	Stomach	HINT1	NES 0.192059	2.0×10^{-6}	
**rs4513689**	eQTLGen Phase I	Blood	CDC42SE2	Z -15.3124	6.30×10^{-53}	
**rs4513689**	eQTLGen Phase I	Blood	LYRM7	Z -6.3089	2.81×10^{-10}	
**rs4513689**	Ensembl VEP	–	–	–	–	Gene: –; most severe consequence: intron_variant; biotype: lncRNA; transcript consequence: intron_variant; non_coding_transcript_variant
**rs73591340**	GTEx v8	Queried GI tissues	No significant eQTL identified	–	–	
**rs73591340**	eQTLGen Phase I	Blood	No significant cis-eQTL identified	–	–	
**rs73591340**	Ensembl VEP	–	–	–	–	Gene: –; most severe consequence: intron_variant; biotype: lncRNA; transcript consequence: intron_variant; non_coding_transcript_variant
**rs817852**	GTEx v8	Queried GI tissues	No significant eQTL identified	–	–	
**rs817852**	eQTLGen Phase I	Blood	RAD23B	Z 4.9793	6.38×10^{-7}	
**rs817852**	Ensembl VEP	–	–	–	–	Gene: –; most severe consequence: intergenic_variant
**rs1925245**	GTEx v8	Transverse colon	PLCE1	NES −0.163633	4.0×10^{-5}	
**rs1925245**	eQTLGen Phase I	Blood	HELLS	Z 5.9976	2.00×10^{-9}	
**rs1925245**	Ensembl VEP	–	–	–	–	Gene: PLCE1; most severe consequence: intron_variant; biotype: nonsense_mediated_decay; protein_coding; transcript consequence: NMD_transcript_variant; intron_variant
**rs77396079**	GTEx v8	Queried GI tissues	No significant eQTL identified	–	–	
**rs77396079**	eQTLGen Phase I	Blood	No significant cis-eQTL identified	–	–	
**rs77396079**	Ensembl VEP	–	–	–	–	Gene: –; most severe consequence: intergenic_variant
**rs78504122**	GTEx v8	Queried GI tissues	No significant eQTL identified	–	–	
**rs78504122**	eQTLGen Phase I	Blood	POLR3E	Z 5.0498	4.43×10^{-7}	

**rs78504122**	eQTLGen Phase I	Blood	RRN3P1	Z 8.3616	6.20×10^{-17}	
**rs78504122**	Ensembl VEP	–	–	–	–	Gene: MOSMO; most severe consequence: intron_variant; biotype: nonsense_mediated_decay; protein_coding; transcript consequence: NMD_transcript_variant; intron_variant
**rs7259854**	GTEx v8	Stomach	ZNF565	NES −0.442788	0.0*	
**rs7259854**	GTEx v8	Stomach	LINC00665	NES 0.203663	2.0×10^{-6}	
**rs7259854**	GTEx v8	Stomach	ZFP14	NES −0.146000	6.6×10^{-5}	
**rs7259854**	GTEx v8	Terminal ileum	ZNF565	NES −0.324219	1.8×10^{-5}	
**rs7259854**	GTEx v8	Sigmoid colon	COX7A1	NES −0.199469	3.0×10^{-6}	
**rs7259854**	GTEx v8	Sigmoid colon	ZNF565	NES −0.292221	7.0×10^{-6}	
**rs7259854**	GTEx v8	Sigmoid colon	LINC00665	NES 0.203595	2.2×10^{-5}	
**rs7259854**	GTEx v8	Transverse colon	ZNF565	NES −0.333699	0.0*	
**rs7259854**	GTEx v8	Transverse colon	LINC00665	NES 0.151303	4.6×10^{-5}	
**rs7259854**	eQTLGen Phase I	Blood	ZFP82	Z 16.0746	3.86×10^{-58}	
**rs7259854**	eQTLGen Phase I	Blood	CTD-3162L10.1	Z 13.1413	1.91×10^{-39}	
**rs7259854**	eQTLGen Phase I	Blood	ZNF565	Z -17.6281	1.50×10^{-69}	
**rs7259854**	eQTLGen Phase I	Blood	LINC00665	Z 26.1899	3.47×10^{-151}	
**rs7259854**	eQTLGen Phase I	Blood	ZFP14	Z -12.8630	7.27×10^{-38}	
**rs7259854**	eQTLGen Phase I	Blood	CAPNS1	Z -7.4436	9.80×10^{-14}	
**rs7259854**	eQTLGen Phase I	Blood	ZNF567	Z 4.4883	7.18×10^{-6}	
**rs7259854**	Ensembl VEP	–	–	–	–	Gene: ZNF565; most severe consequence: non_coding_transcript_exon_variant; biotype: protein_coding; retained_intron; transcript consequence: intron_variant; non_coding_transcript_exon_variant
**rs3026502**	GTEx v8	Queried GI tissues	No significant eQTL identified	–	–	
**rs3026502**	eQTLGen Phase I	Blood	No significant cis-eQTL identified	–	–	
**rs3026502**	Ensembl VEP	–	–	–	–	Gene: –; most severe consequence: intergenic_variant
**rs77819704**	GTEx v8	Queried GI tissues	No significant eQTL identified	–	–	
**rs77819704**	eQTLGen Phase I	Blood	No significant cis-eQTL identified	–	–	
**rs77819704**	Ensembl VEP	–	–	–	–	Gene: –; most severe consequence: intergenic_variant

GTEx v8 was used to assess eQTL associations in gastrointestinal tissues, including stomach, terminal ileum, sigmoid colon, and transverse colon. eQTLGen Phase I entries represent significant cis-eQTL associations from blood-based expression data. Ensembl VEP was used to annotate predicted variant consequences. NES, normalized effect size; eQTL, expression quantitative trait locus; VEP, Variant Effect Predictor. *P-values shown as 0.0

Supplementary Table S7. AUCs and optimism-corrected AUCs from bootstrap internal validation of multivariable models

	Predictors	AUC	Optimism	Optimism-corrected AUC
Model 1	Age, sex, BMI	0.6649	0.0388	0.6262
Model 2	Age, sex, BMI, laboratory parameters	0.9004	0.0435	0.8570
Model 3	Age, sex, BMI, medications	0.7757	0.0416	0.7341
Model 4	Age, sex, BMI, SNP	0.7589	0.0269	0.7319
Model 5	Age, sex, BMI, medications, laboratory parameters, SNP	0.9524	0.0386	0.9138

AUC, area under the receiver operating characteristic curve; BMI, body mass index; SNP, single-nucleotide polymorphism. Medications included concomitant anti-arthritic and gastrointestinal medications. Laboratory parameters included hemoglobin, neutrophil count, albumin, bilirubin, white blood cell count, and erythrocyte sedimentation rate. The SNP included in Models 4 and 5 was rs1925245. Internal validation was performed using 1,000 bootstrap resamples; optimism-corrected AUCs were calculated by subtracting the estimated optimism from the apparent AUC.

Supplementary Figure S5. ROC curves of the five exploratory discrimination models.

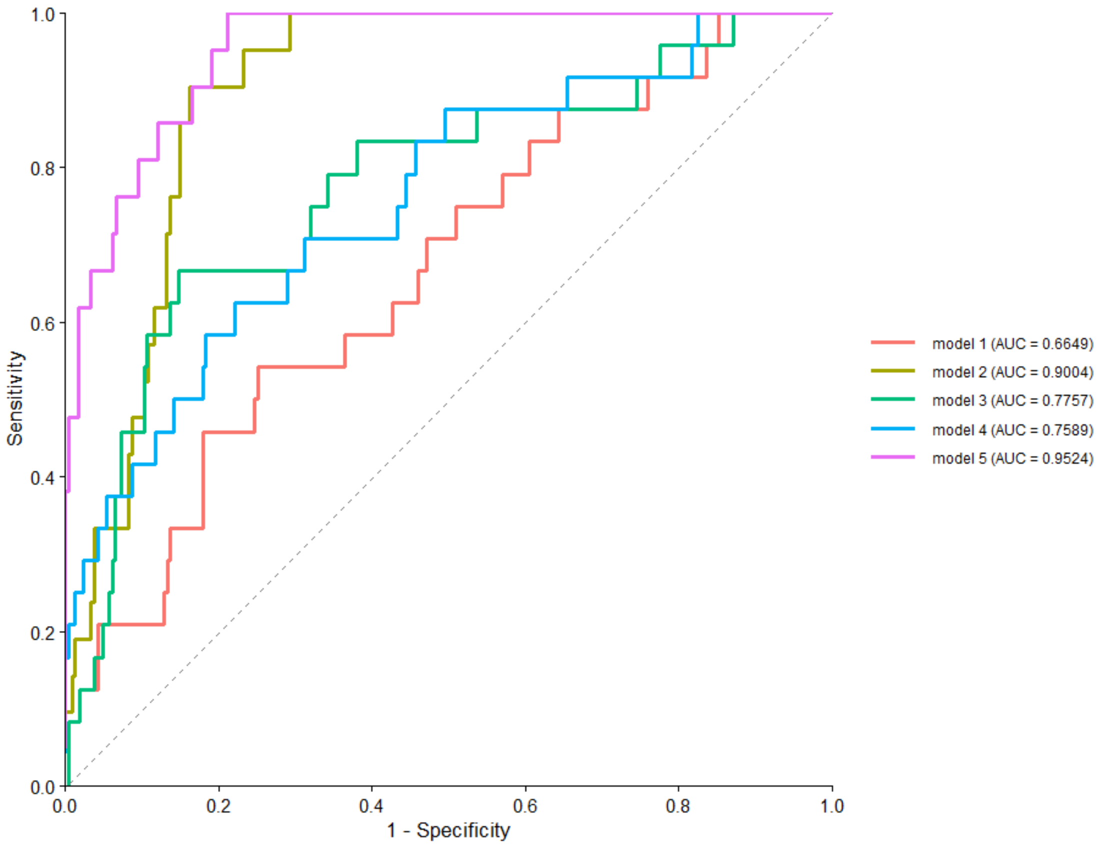

Receiver operating characteristic (ROC) curves showing the discriminatory performance of five models. Model 1 included age, sex, and BMI; Model 2 additionally included laboratory parameters; Model 3 additionally included concomitant medications; Model 4 included age, sex, BMI, and rs1925245; and Model 5 included age, sex, BMI, laboratory parameters, concomitant medications, and rs1925245. The apparent area under the curve (AUC) for each model is shown in the legend. BMI, body mass index; AUC, area under the curve.
